# SARS-CoV-2 associated unilateral parotitis in children: A case report and literature review

**DOI:** 10.3892/br.2024.1771

**Published:** 2024-03-27

**Authors:** Andrea Marino, Giovanni Cacciaguerra, Serena Spampinato, Monica Palermo, Arturo Biasco, Emanuele Liotta, Salvatore Cocuzza, Emmanuele Venanzi Rullo, Giuseppe Nunnari, Piero Pavone

**Affiliations:** 1 Department of Clinical and Experimental Medicine, Unit of Infectious Diseases, University of Catania, ARNAS Garibaldi Hospital, I-95122 Catania, Italy; 2 Department of Clinical and Experimental Medicine, Section of Paediatrics and Child Neuropsychiatry, School of Specialization in Paediatrics, University of Catania, I-95124 Catania, Italy; 3 Department of Clinical and Experimental Medicine, University of Messina, I-98124 Messina, Italy; 4 Department of Medical Surgical Sciences and Advanced Technologies ‘GF Ingrassia’, Radiology Unit 1, University of Catania, I-95125 Catania, Italy; 5 Department of Medical and Surgical Sciences and Advanced Technologies ‘GF Ingrassia’, ENT Section, University of Catania, I-95124 Catania, Italy

**Keywords:** parotitis, Severe acute respiratory syndrome coronavirus 2 parotitis, atypical Coronavirus disease 2019, Coronavirus disease 2019 in children

## Abstract

Severe acute respiratory syndrome coronavirus 2 (SARS-CoV-2) infection usually affects the respiratory system; however, a number of atypical manifestations of this disease have also been reported, especially in children. The present study reports a case of a 12-year-old presenting with right unilateral parotitis and sialadenitis and SARS-CoV-2 infection. The young patient, after a 3-day history of fever, was brought to our clinic (Polyclinic University Hospital ‘G. Rodolico’, Catania, Italy) for the sudden onset of unilateral parotitis accompanied by sialadenitis and hyperaemia of the skin, which was tender to touch. The SARS-CoV-2 molecular swab was positive; the ultrasound of the affected region showed an increase in the volume of the parotid and sublingual gland and reactive lymph nodes compatible with parotitis and sialadenitis. This case suggests that, in the present Coronavirus disease 2019 pandemic, SARS-CoV-2 should be included in the differential diagnosis of parotitis and sialadenitis along with mumps and flue. Notably, a respiratory panel and serology for other potential causes are needed in case of parotitis-like disease.

## Introduction

SARS-CoV-2, the virus responsible for the global pandemic of Coronavirus Disease 2019 (COVID-19), first identified in Wuhan, China, in December 2019, has led to unprecedented challenges in public health, clinical management and medical research. This coronavirus primarily targets human cells from several tissues by binding to the angiotensin-converting enzyme II (ACE2) receptor (1). The virus predominantly spreads through respiratory droplets that are released during activities such as coughing, sneezing or even talking, explaining the high transmissibility and infectious nature of the virus (2-4).

The clinical spectrum of COVID-19 is remarkably diverse, ranging from asymptomatic or mild flu-like symptoms in ~70% of patients, to severe respiratory distress and multi-organ failure in more critical cases (2). Commonly reported symptoms include fever, malaise, dry cough and sore throat. However, the pandemic has unveiled a myriad of atypical presentations that deviate from the classical respiratory symptoms, thereby complicating the diagnostic process (5). These atypical manifestations include, but are not limited to, ocular symptoms, cardiac complications such as myocarditis, dermatological signs, gastrointestinal and hepatic disturbances, cerebrovascular incidents and various neurological symptoms (6).

The recognition of these unusual presentations is pivotal in ensuring timely diagnosis and appropriate management of COVID-19. Such awareness also aids in implementing effective isolation measures to curtail the spread of SARS-CoV-2. Among these atypical manifestations, oral symptoms have garnered attention, ranging from simple taste disturbances to more complex conditions such as mucosal lesions and salivary gland infections (7).

The impact of SARS-CoV-2 on children, although initially perceived as minor, has evolved into a subject of major concern. In paediatric patients, the virus often manifests in forms markedly different from adults (from asymptomatic diseases, which represent the most common form, to multisystem inflammatory syndrome), which can lead to underdiagnosis or misdiagnosis. This is particularly crucial as children, being active social agents in schools and family units, can be key vectors in the transmission of the virus. Furthermore, the emergence of multisystem inflammatory syndrome in children associated with COVID-19 underscores the unpredictable nature of the virus in the younger population (8). Atypical manifestations in children can be more cumbersome than those involving adults, resulting in differential diagnosis and clinical management that may pose challenges for clinicians (9).

As such, expanding our knowledge on the diverse clinical presentations of COVID-19 in children, such as the case of SARS-CoV-2 induced parotitis presented in the present case, is not only pivotal for the paediatric care but also for the broader public health strategy. This case report aims to shed light on the less explored aspects of COVID-19 in paediatrics, illustrating how even non-respiratory symptoms can herald the presence of the virus in this demographic, thus necessitating a broader clinical vigilance among healthcare providers.

The present case report contributed to this growing body of literature by reporting SARS-CoV-2 infection presenting primarily with unilateral parotitis and sialadenitis in a 12-year-old girl, further expanding our understanding of the virus's clinical heterogeneity.

## Case report

In November 2023, a 12-year-old female patient visited our clinic (Polyclinic University Hospital ‘G. Rodolico’, Catania, Italy) due to the sudden onset of right-sided unilateral parotitis, accompanied by sialadenitis, hyperaemia of the skin and pain upon touch. She was up-to-date on her immunizations, including the measles-mumps-rubella vaccine. The patient had not previously been vaccinated for SARS-CoV-2. The parents of the patient reported no history of taking medications such as propylthiouracil, phenothiazines, iodides or phenylbutazone. The patient had no chronic diseases. Upon admission, her temperature was 36.3˚C, heart rate was 78 beats per minute, blood pressure was 110/69 mmHg and oxygen saturation in room air was 99%. The parents of the patient reported intermittent fevers (up to 38˚C) for 3 days prior to admission without respiratory symptoms or facial swelling. Fever was treated with paracetamol.

Clinical examination was normal for the age and sex of the patient. Blood tests revealed in range haemoglobin and platelet counts. The total white count was 8,440 cells/ml, with neutrophils at 6,380 cells/ml (75.6%) and lymphocytes at 1,588 cells/ml (18.8%). C-reactive protein levels were raised (15 mg/dl; normal range, 0-0.5 mg/dl) as well as erythrocyte sedimentation rate (47 mm/h; normal range, <10 mm/h), ferritin and lactate dehydrogenase levels were within the normal range.

Mumps serology was negative for IgM and positive for IgG. The main viral infectious disease screenings, including serology and a respiratory panel on a pharyngeal swab (BioFire^®^ Respiratory 2.1 Panel; BioFire Diagnostics, Inc.; bioMérieux), were negative. This included tests for Epstein-Barr virus, human herpesvirus 6, human immunodeficiency virus, respiratory syncytial virus, parainfluenza virus types 2 and 3, influenza A and B viruses, adenovirus, coxsackie viruses, parvovirus B19, lymphocytic choriomeningitis virus, human bocavirus and paramyxovirus RNA. Overall, two sets of blood cultures were negative. A nasopharyngeal swab tested positive for SARS-CoV-2, which was subsequently confirmed by the BioFire^®^ respiratory panel. A chest radiograph showed no parenchymal lung alterations suggestive of a pneumonic process. The patient had no upper or lower respiratory symptoms.

**Figure 1:**
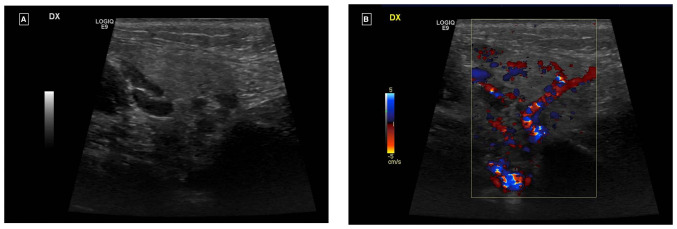
(A) Ultrasound and (B) Ecocolor-Doppler examination of the right parotid gland.

Ultrasound examination revealed an enlarged right parotid gland and right submandibular gland, both with a homogeneous echostructure and a diffused increase in echogenicity of the glandular parenchyma. There was also a diffuse increase in intraparenchymal vascularity and reactive adenitis. No fluid collection, obstructing stones, dilatation of salivary ducts, masses or abscesses were observed (Fig. 1A and B).

Non-steroidal anti-inflammatory drugs therapy (NSAID) with ibuprofen was started at a dosage of 200 mg every 12 h. Although clinical symptoms did not significantly improve, within 48 h clear saliva without pus was observed to discharge from the parotid glands. The patient was discharged with a prescription for analgesic therapy to be used as needed. SARS-CoV-2 swab tested negative after 7 days and the patient did not develop other symptoms or manifestations. Parents reported they administered ibuprofen for a total of 6 days.

At a follow-up visit 2 weeks later, we noted the complete resolution of the clinical condition of the patient.

## Discussion

The present case highlights the novelty of SARS-CoV-2-associated unilateral parotitis in a 12-year-old girl, a rare presentation within the paediatric population. Diverging from typical respiratory symptoms of COVID-19, this instance emphasizes the capability of the virus to cause glandular manifestations such as parotitis. The effective management with NSAIDs, without preceding respiratory symptoms or common viral infections, underscores the importance of considering COVID-19 in differential diagnoses for acute parotitis in children, expanding our understanding of the virus's diverse clinical manifestations.

Similar cases have emerged in the literature since the onset of the COVID-19 pandemic, predominantly in adult populations (10-12). The clinical spectrum of SARS-CoV-2 infection typically ranges from minimally symptomatic disease to severe pneumonia and critical illness, with ~70% of patients either asymptomatic or exhibiting mild flu-like symptoms, such as fever, dry cough and sore throat (9).

The atypical presentations of SARS-CoV-2 infection are diverse, including myocarditis, stroke, liver damage, gastrointestinal symptoms, ocular manifestations, dermatological lesions and a range of neurological symptoms, observed in both mild and severe cases (13).

The parotitis seen in SARS-CoV-2 infections can be attributed to several interacting pathological mechanisms (11,12). Direct viral invasion is a primary suspect, where SARS-CoV-2 infiltrates the salivary gland epithelium by binding to ACE2 receptors, causing local inflammation and cellular damage (14). Concurrently, the host's immune response, while attempting to combat the virus, may overshoot, releasing a deluge of cytokines in a detrimental ‘cytokine storm’, leading to further tissue inflammation and gland swelling (15,16). Compounding this, SARS-CoV-2 affects endothelial cells, causing dysfunction that manifests as increased vascular permeability and oedema, which is a condition conducive to parotitis (17). The propensity of the virus to induce a hypercoagulable state could also precipitate microthrombi within the tiny blood vessels of the salivary glands, impairing blood flow and oxygen delivery, exacerbating inflammation (18). Moreover, the concept of molecular mimicry may offer insight into prolonged or recurrent gland inflammation, as the immune system's production of antibodies against the virus could inadvertently target structurally similar antigens within the salivary glands (19). This complex interplay of direct viral effects, immune response dysregulation, vascular pathology and autoimmune phenomena not only elucidates the occurrence of parotitis in COVID-19 but also reflects the multisystemic impact of SARS-CoV-2, warranting a broad spectrum of considerations in the clinical management of these patients (2,20,21).

The timeline for the development of parotitis during SARS-CoV-2 infection is not well-defined. In several reports, parotitis was noted 1-3 days after the onset of coronavirus symptoms (11,22,23). Parotitis not related to mumps has been associated with various viruses, including enteroviruses, adenoviruses, cytomegalovirus, influenza, parainfluenza, Epstein-Barr, herpes simplex and herpes virus 6(24).

In the present case, the patient presented with acute mumps-like symptoms and unilateral sialadenitis following a 3-day history of fever, without any chronic diseases, trauma and with normal blood tests. The positive SARS-CoV-2 swab, combined with ultrasound findings which ruled out oncological or obstructive conditions, along with normal blood samples and physical examination, facilitated a non-invasive approach. This approach allowed us to reassure the parents and monitor the patient over time, avoiding the immediate use of more invasive imaging techniques such as CT scans, which involve ionizing radiation.

To the best of our knowledge the scientific literature report before the present study reports only six patients who developed a parotitis which could be ascribed to SARS-CoV-2 infection (Table I). The age of the patient in the present case is somewhat aligned with the range observed in the literature, with reported cases varying from 2 months to 7 years of age (25,26). While most cases involved males, the present patient is female, suggesting no strong sex predilection for SARS-CoV-2 associated parotitis.

Unlike other cases, the present patient had no preceding respiratory symptoms, which is atypical given that respiratory symptoms were present in most literature cases, albeit mild in some (27), highlighting the variable presentation of the disease. All patients had undergone neck ultrasounds. The laboratory parameters in the current case were mostly within normal ranges, contrasting with other reports where leucocytosis and elevated acute-phase reactants were common (28). This discrepancy may point to a less aggressive inflammatory response in the present patient.

Our patient was treated only with NSAID therapy, without the need of either antibiotics (often overprescribed by clinicians aiming to prevent bacterial superinfections) or corticosteroid therapy.

The pathogenesis of SARS-CoV-2-associated sialadenitis is yet to be fully understood. However, SARS-CoV-2 should be considered in the differential diagnosis of parotitis/sialadenitis, particularly if it presents unilaterally. Prompt isolation measures are crucial to mitigate the spread of infection (29).

The epidemiological landscape of COVID-19 in the paediatric population presents a multifaceted challenge. Although children account for a significant portion of total cases, their clinical manifestations often differ from adults, leading to unique considerations in public health strategies (30). Notably, the prevalence of COVID-19 among children varies with age, showing higher incidence in school-aged children and adolescents compared with younger children (31,32). Additionally, the impact of socioeconomic factors is pronounced in this demographic, as children from lower socioeconomic backgrounds face increased exposure risks and potential health disparities (33). Understanding these dynamics is crucial, not only for direct paediatric care but also for broader community health policies, especially considering the role of children in viral transmission within schools and households (34). This complex epidemiological profile underscores the need for tailored approaches in both prevention and treatment strategies for COVID-19 among children.

The present case underscores the importance of including SARS-CoV-2, and coronaviruses in general, in the list of pathogens responsible for parotitis and sialadenitis, alongside mumps and influenza. Notably, a comprehensive respiratory panel and serology are essential for accurate diagnosis in cases presenting with parotitis-like symptoms.

Effective COVID-19 prevention in children hinges on a multi-faceted approach that balances public health guidelines with the unique needs of the paediatric population. Vaccination, when available and approved for specific age groups, plays a crucial role in reducing transmission and severity of the disease among children (35).

In conclusion, the present case highlights the need for heightened vigilance and a broader diagnostic perspective during the ongoing COVID-19 pandemic. Understanding the diverse clinical manifestations of SARS-CoV-2, including rare presentations such as unilateral parotitis, is crucial for prompt diagnosis, effective patient management and prevention of virus transmission.

## Figures and Tables

**Table I:** Characteristics of SARS-CoV-2 associated parotitis in children.

Author	No. of patients	Age	Sex	Symptoms	Lab findings	SARS-CoV-2 test performed	Respiratory symptoms	Treatment	Duration (days)	Outcome	(Refs.)
Brehm *et al*	1	10 weeks	M	Facial swelling, decreased appetite	Leucocytosis, high platelets	PCR	Rhinorrhoea, cough	Amoxicillin/clavulanic acid	7	Resolved	(27)
Likitnukul	1	4 years	M	Facial swelling, low grade fever, decreased appetite	High CRP, high amylase	PCR	Mild cough	Analgesic (not specified)	3	Resolved	(28)
Keles *et al*	1	4 years	M	Facial swelling and pain, difficulty swallowing	High amylase	PCR	None	NSAID (not specified)	3	Resolved	(26)
Sharma *et al*	3	7 years	F	Facial swelling, partial maloc- clusion, trismus, decreased appetite	High CRP and ESR, high amylase	PCR	Cough (4 weeks before)	Prednisolone 1 mg/kg/day	5	Resolved	(25)
		3.5 years	M	Facial swelling, fever, poor appetite	Leucocytosis, high ESR and CRP	PCR	None	Prednisolone 1 mg/kg/day	5	Resolved	
		2 months	M	Facial swelling, irritability, decreased appetite, fever	Leucocytosis, high CRP	PCR	None	Metilpredni- solone 2 mg/kg/day	5	Resolved	

SARS-CoV-2, severe acute respiratory syndrome coronavirus 2; CRP, C reactive protein; NSAID, non-steroidal anti-inflammatory drugs; ESR, erythrocyte sedimentation rate.

## Data Availability

Data sharing is not applicable to this article as no datasets were generated or analysed during the current study.
